# Liver-specific Repin1 deficiency impairs transient hepatic steatosis in liver regeneration

**DOI:** 10.1038/s41598-018-35325-3

**Published:** 2018-11-15

**Authors:** Kerstin Abshagen, Bastian Degenhardt, Marie Liebig, Anna Wendt, Berit Genz, Ute Schaeper, Michael Stumvoll, Ute Hofmann, Marcus Frank, Brigitte Vollmar, Nora Klöting

**Affiliations:** 1 Institute for Experimental Surgery, University Medicine Rostock, Schillingallee 69a, 18057 Rostock, Germany; 2 QIMR Berghofer Medical Research Institute, 200 Herston Road, Herston, QLD 4006 Australia; 3 Silence Therapeutics GmbH, Berlin, Robert Rössle Strasse 10, 13125 Berlin, Germany; 4 Department of Medicine, University of Leipzig, Liebigstrasse 18, 04103 Leipzig, Germany; 5 Dr. Margarete Fischer-Bosch Institute of Clinical Pharmacology, Auerbachstrasse 112, 70376 Stuttgart and University of Tuebingen, Tuebingen, Germany; 6 Medical Biology and Electron Microscopy Centre, University Medicine Rostock, Strempelstrasse 14, 18057 Rostock, Germany; 7 Integrated Research and Treatment Center (IFB) Adiposity Diseases, University of Leipzig, Liebigstrasse 19-21, 04103 Leipzig, Germany

## Abstract

Transient hepatic steatosis upon liver resection supposes functional relationships between lipid metabolism and liver regeneration. Repin1 has been suggested as candidate gene for obesity and dyslipidemia by regulating key genes of lipid metabolism and lipid storage. Herein, we characterized the regenerative potential of mice with a hepatic deletion of Repin1 (LRep1−/−) after partial hepatectomy (PH) in order to determine the functional significance of Repin1 in liver regeneration. Lipid dynamics and the regenerative response were analyzed at various time points after PH. Hepatic Repin1 deficiency causes a significantly decreased transient hepatic lipid accumulation. Defects in lipid uptake, as analyzed by decreased expression of the fatty acid transporter Cd36 and Fatp5, may contribute to attenuated and shifted lipid accumulation, accompanied by altered extent and chronological sequence of liver cell proliferation in LRep1−/− mice. *In vitro* steatosis experiments with primary hepatocytes also revealed attenuated lipid accumulation and occurrence of smaller lipid droplets in Repin1-deficient cells, while no direct effect on proliferation in HepG2 cells was observed. Based on these results, we propose that hepatocellular Repin1 might be of functional significance for early accumulation of lipids in hepatocytes after PH, facilitating efficient progression of liver regeneration.

## Introduction

Initially identified as a candidate gene in the quantitative trait locus for facets of the metabolic syndrome in subcongenic rat strains^1^, further investigations showed that expression of the Replication initiator 1 (Repin1) in the liver and adipose tissue is significantly associated with obesity and dyslipidemia^2–5^. Repin1 is ubiquitously expressed with highest amounts in liver and intraabdominal adipose tissue^1^. Ruschke *et al*.^2^ showed that Repin1 in adipocytes regulates the expression of genes involved in adipogenesis, lipid droplet formation and fusion as well as glucose and fatty acid (FA) transport. Moreover, beneficial physiological consequences of a liver-restricted Repin1 deficiency (LRep1−/−), as lower body weight, reduced hepatic steatosis, increased energy expenditure and physical activity as well as improved insulin sensitivity, underline the significant role of Repin1 in glucose homeostasis and lipid metabolism^6^.

Altered regulation of metabolic genes and pathways involved in insulin signaling, lipid and glucose metabolism is functionally important for the initiation of regeneration upon hepatic insufficiency. Thereby, transient accumulation of lipids in the regenerating liver is a well-known phenomenon and appears to be essential for adequate liver regeneration after hepatic resection^7–10^. It might serve as energy source for subsequent metabolic events associated with the regenerative process and reconstruction of cell membranes^11^. Rapid increase in hepatic triglycerides (TG) results from enhanced lipolysis in peripheral adipose tissue and influx of non-esterified FAs into the liver^12^.

The significance of hepatic fat for liver regeneration is supported by multiple studies, showing that reduced hepatic adipogenesis^13,14^, disruption/inhibition of ß-oxidation^15–17^ or defects in efficient lipid uptake, transport and formation^18,19^ are substantially associated with inhibition of the regenerative response of the organ. However, some reports showed the opposite^11,20^ and thus, it is still controversially discussed whether fat or rather glucose is the main energy substrate after hepatectomy^15,21,22^. It is supposed that fat is the main energy substrate for liver regeneration only very early after resection, if glucose is not available. Furthermore, it is needed primarily by hepatocytes of acinar zone 1 which prefer fat as energy source^21,22^. Nevertheless, the complex mechanisms that regulate initiation and resolution of transient hepatic steatosis upon resection and the functional significance of these events for liver regeneration are not fully clarified.

On the contrary, there is considerable evidence that fatty livers are suboptimal for surgical resection or transplantation, predominantly because of their increased susceptibility to ischemia-reperfusion injury and their disrupted regenerative response^23–25^. However, depending on the experimental model used, discrepant data regarding the regenerative capacity of steatotic livers exist^26–30^.

Based on previous findings in LRep1−/− mice regarding altered hepatic lipid storage, we hypothesized that Repin1 plays a functional role in hepatic regeneration. Therefore, we investigated the regenerative potential of LRep1−/− after partial hepatectomy (PH).

## Results

### Hepatic Repin1 expression upon liver resection

**Figure 1:**
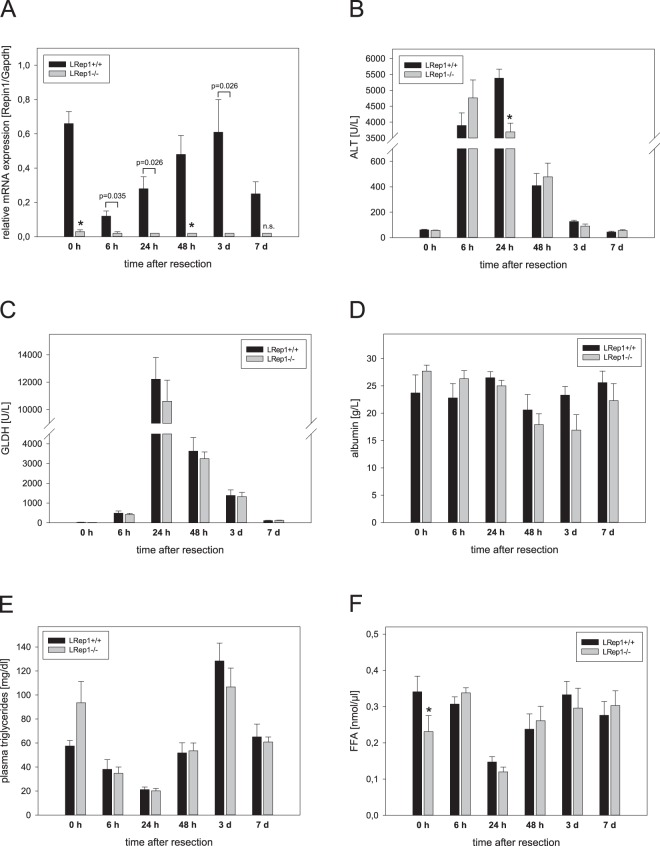
(**A**) Quantitative RT-PCR analysis of Repin1 mRNA expression in liver tissue of LRep1+/+ and LRep1−/− mice at different time points after PH (n = 6–7 per genotype and time point). Values are given as means ± SEM. Significance of differences between the groups of an individual time point was tested using Mann-Whitney Rank Sum test followed by Bonferroni correction (*p < 0.0083 vs. LRep1+/+ of the individual time point). Analysis of plasma activities of (**B**) alanine aminotransferase (ALT) and (**C**) glutamate dehydrogenase (GLDH) as well as (**D**) plasma albumin concentration in LRep1+/+ and LRep1−/− mice at different time points after PH (n = 5–8 per genotype and time point). Analysis of (**E**) TGs and (**F**) free FAs in plasma of LRep1+/+ and LRep1−/− mice at different time points after PH (n = 6–8 per genotype and time point). Significance of differences between the groups of an individual time point was tested using two way ANOVA, *p < 0.05 vs. LRep1+/+ 0 h).

Quantitative RT-PCR analysis revealed almost no mRNA expression of *Repin1* in LRep1−/− compared to wildtype mice, in which Repin1 level transiently decreased immediately after PH (Fig. 1A). Non-parenchymal cells were responsible for the remaining low *Repin1* expression in LRep1−/− mice.

### Liver injury and liver function

Resection-associated liver injury, as given by a transient rise of liver enzymes upon PH, was significantly diminished in LRep1−/− animals with ALT values being about 1/3 lower than in LRep1+/+ plasma 24 h after PH (Fig. 1B). GLDH levels were also increasing upon 70% resection with a peak observed at 24 h, but with no significant differences between both genotypes (Fig. 1C). In contrast to that, albumin synthesis was maintained over the whole observation period with a minor decline in LRep1−/− mice at 72 h after PH (Fig. 1D).

### Systemic and hepatic lipid profile

**Figure 2:**
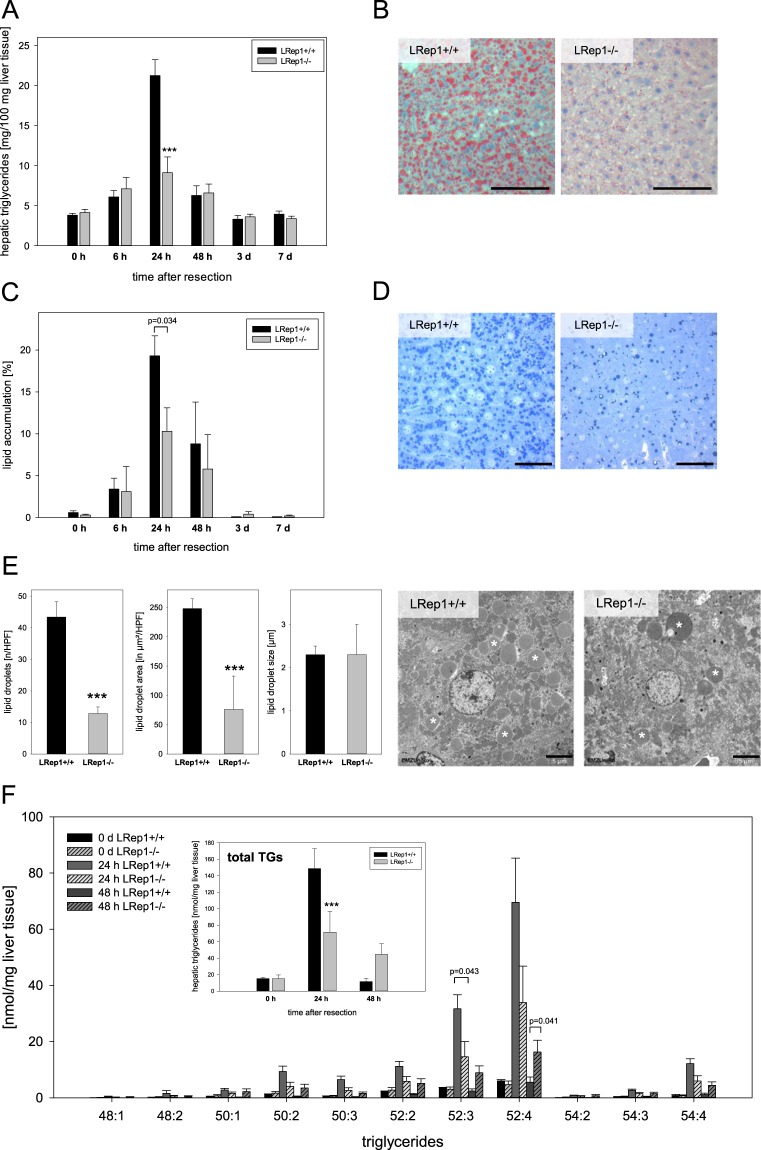
(**A**) Quantification of TGs in liver tissue of LRep1+/+ and LRep1−/− mice at different time points after PH using a standard kit (Values are given as means ± SEM; n = 6–8 per genotype and time point, Mann-Whitney Rank Sum test followed by Bonferroni correction, ***p < 0.001 vs. LRep1+/+ 24 h). (**B**) Representative Oil Red O stained frozen liver sections of LRep1+/+ and LRep1−/− mice 24 h after PH. Scale bars represent 100 µm. (**C**) Histomorphometric analysis of semi-thin toluidine blue stained liver sections to quantify fat deposition as percentage of blue stained area compared with the total section area. Values are given as means ± SEM (n = 3–6 per genotype and time point; Mann-Whitney Rank Sum test followed by Bonferroni correction). (**D**) Representative semi-thin toluidine blue stained liver sections of LRep1+/+ and LRep1−/− mice 24 h after PH. Scale bars represent 100 µm. (**E**) Quantitative analysis of the number, area and size of lipid droplets in hepatocytes of LRep1+/+ and LRep1−/− mice 24 h after PH by means of transmission electron microscopy and representative images (lipid droplets are marked with white asterisks). Scale bars represent 5 µm. Values are given as means ± SEM (n = 5 per genotype; t- test, ***p < 0.001 vs. LRep1+/+). (**F**) Quantitative analysis of TG groups as well as total amount of liver TGs (insert) at baseline (0 h), 24 and 48 h after PH in LRep1+/+ and LRep1−/− mice using LC-MS. The first number is equivalent to the carbon numbers and the second number represents the numbers of double bonds. Values are given as means ± SEM (n = 6 per genotype and time point; total TGs: two way ANOVA, ***p < 0.001 vs. LRep1+/+; TG groups: Mann-Whitney Rank Sum test followed by Bonferroni correction).

Hepatic Repin1 deficiency caused dyslipidemia in plasma at baseline (Fig. 1E and F) and altered hepatic lipid content after PH (Fig. 2). Whereas LRep1−/− mice showed higher plasma TG levels at baseline (Fig. 1E), liver resection initially induced a decrease of plasma TGs in both genotypes with lowest levels being present at 24 h. Afterwards systemic TGs were increasing again. A similar trend after PH with no significant differences between both mouse strains was observed for plasma FFA, in which a drop down of FFA has been seen at 24 h (Fig. 1F).

Liver TGs and lipids were analyzed by different methods. Biochemical quantification of hepatic TG demonstrated a PH-induced steep rise in TG of LRep1+/+ livers with a peak value 24 h after PH (Fig. 2A). In contrast, livers of LRep1−/− revealed significantly reduced TG at 24 h after PH (Fig. 2A). Additionally, Oil red O stained frozen liver sections (Fig. 2B) showed also a much lesser extent of fat positive area in LRep1−/− tissue. Moreover, fat deposition was quantified by histomorphometric analysis of semi-thin toluidine blue stained liver sections (Fig. 2C). This analysis also confirmed the dramatic increase of hepatic lipids 24 h after resection in wildtype mice, whereas lipid accumulation was much less pronounced in LRep1−/− mice. This means that 24 h after PH livers of LRep1+/+ mice consisted of ~20% lipid droplets compared to ~10% in LRep1−/− tissue, whereas livers of sham mice (0 h) simply contained <1% lipids (Fig. 2C). Representative toluidine blue stained liver sections (Fig. 2D) display this distinct difference in fat content between both genotypes at 24 h. Further electron microscopic analysis of the lipid droplets (Fig. 2E) revealed a significantly reduced number and total area of lipid droplets in LRep1−/− compared to LRep1+/+ hepatocytes 24 h after PH, with no difference in droplet size. Representative electron microscopic images displaying the higher number of lipid droplets in wildtype livers compared to LRep1−/− tissue (Fig. 2E, white asterisks).

For the time points with the most striking differences in lipid content between both genotypes, we analyzed the pattern of TG subgroups using LC-MS (Fig. 2F). Generally, the most prominent TG groups during early liver regeneration were TG 52:3 and 52:4. Compared to wildtype LRep1−/− mice exhibited markedly lower levels of these TG groups at 24 h after resection, but showed a delayed increase 48 h after resection, when in wildtype livers the levels of these TG groups have already declined. Measurement of total TG levels with this method confirmed the above mentioned results of significantly lower levels in LRep1−/− compared to LRep1+/+ livers at 24 h after resection. In LRep1−/− mice a delayed increase of TG at 48 h (Fig. 2F, inserted graph) was detected.

### Hepatic glycogen content

**Figure 3:**
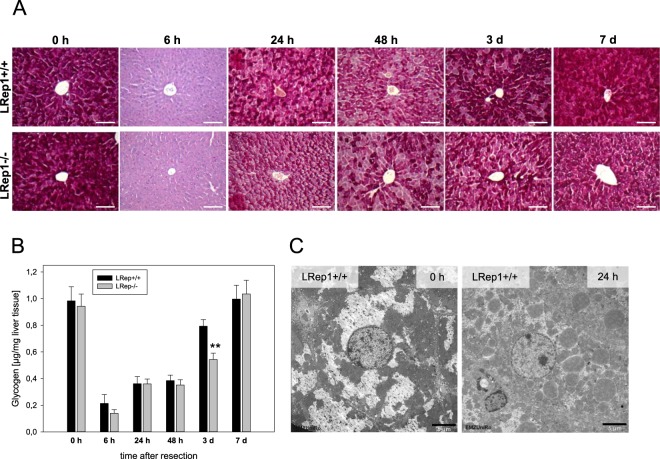
(**A**) Representative images of PAS-stained liver sections of in LRep1+/+ and LRep1−/− mice at different time points after PH. Scale bars represent 100 µm. (**B**) Quantitative analysis of liver glycogen content in LRep1+/+ and LRep1−/− mice at different time points after PH. Values are given as means ± SEM (n = 6–8 per genotype and time point). Significance was tested by two-way ANOVA followed by the appropriate post hoc comparison (Holm-Sidak method), **p < 0.01 vs. LRep1+/+ 3 d. (**C**) Transmission electron microscopic images of LRep1+/+ under physiological conditions (0 h) and 24 h after PH showing loss of glycogen (0 h, white deposition) upon PH. Scale bars represent 5 µm.

As the liver plays an important role in glucose homeostasis and storage, we evaluated the kinetics of glycogen content upon resection by different methods. Analysis of glycogen in liver tissue homogenates revealed a rapid resection-induced loss of glycogen storage in both mouse strains, with almost no existing glycogen at 6 h after PH (Fig. 3A and B). Thereafter, glycogen content replenished slowly and was fully restored 7 d after PH. This could also be verified by PAS staining of LRep1−/− and LRep1+/+ livers (Fig. 3A), impressively showing the initial loss and continuous increase of glycogen after PH. Hepatic Repin1 deficiency was accompanied by a significantly reduced glycogen content at 3 d after PH (Fig. 3B). Representative electron microscopic images of LRep1+/+ also display high glycogen content (white crystalline structures) at basal (0 h) and almost no glycogen deposition 24 h after PH (Fig. 3C).

### Regenerative capacity

**Figure 4:**
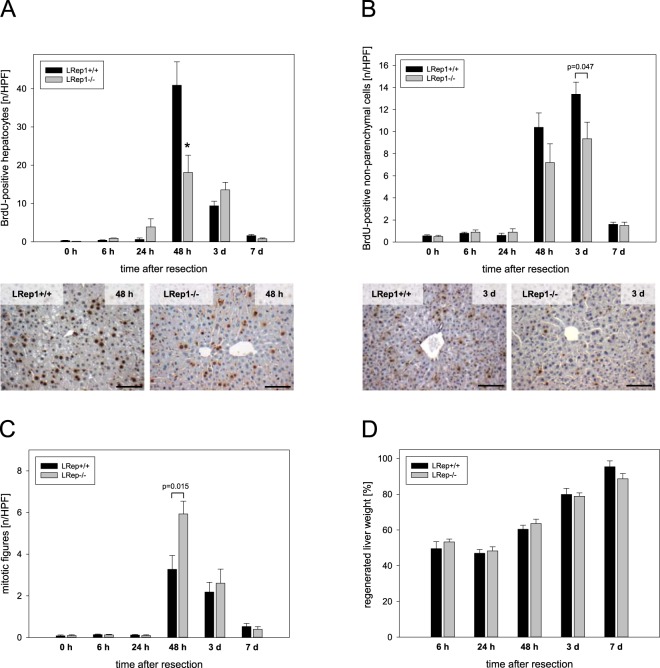
Quantitative analysis of proliferation by means of (**A**) BrdU-positive hepatocytes, (**B**) BrdU-positive non-parenchymal cells and (**C**) mitotic figures (each given as cells/HPF) in livers of LRep1+/+ and LRep1−/− mice at different time points after PH. Values are given as means ± SEM (n = 6–8 per genotype and time point; *p < 0.0083 vs. LRep1+/+ of the individual time point, Mann-Whitney Rank Sum test followed by Bonferroni correction). Representative immunohistochemical images (lower panel) of hepatic tissue in LRep1+/+ and LRep1−/− 48 h (**A**) as well as 3 days (**B**) after resection, displaying the marked rise of BrdU-positive cells, particularly in LRep1+/+, as characteristic sign of cell proliferation in the regenerating liver. Scale bars represent 100 µm. (**D**) Percent regenerated liver weight relative to preoperative liver weight in livers of LRep1+/+ and LRep1−/− mice at different time points after PH. The weight of the regenerated liver was used to calculate the growth of residual liver lobes as ratio of regenerated liver/preoperative liver weight × 100 (%). Pre-operative liver weight was assumed to be 4.7% of body weight for LRep1−/− and 5.0% of body weight for LRep1+/+.

Quantitative analysis of BrdU-stained hepatocytes (Fig. 4A) and non-parenchymal cells (Fig. 4B) as well as counting of mitotic figures (Fig. 4C) were performed as indices of hepatocellular regenerative response upon PH. Livers of LRep1-/- displayed attenuated cell proliferation, as indicated by significantly reduced BrdU incorporation in hepatocytes 48 h after PH compared to LRep1+/+ mice (Fig. 4A,B). Representative immunohistochemical images of hepatic tissue in LRep1+/+ and LRep1−/− livers at 48 h (Fig. 4A) and 3 d (Fig. 4B) after PH demonstrate the high number of BrdU-positive cells as characteristic sign of both parenchymal and non-parenchymal cell proliferation in the regenerating LRep1+/+ liver, whereas livers of LRep1−/− showed less BrdU-positive cells.

To characterize proliferation in more detail, hepatocytes with mitotic figures were counted at the indicated time points (Fig. 4C). Interestingly, 48 h after resection LRep1−/− showed a markedly higher number of hepatocytes being in mitosis compared to LRep1+/+.

There was a constant increase of liver weight upon PH in both genotypes with return to almost pre-operative values (Fig. 4D), with LRep1−/− genotype being associated with a slightly lower liver weight (88.7% ± 2.9%) at day 7 after PH compared to wildtype (95.4% ± 3.3%).

### Hepatic lipogenesis and lipolysis

**Figure 5:**
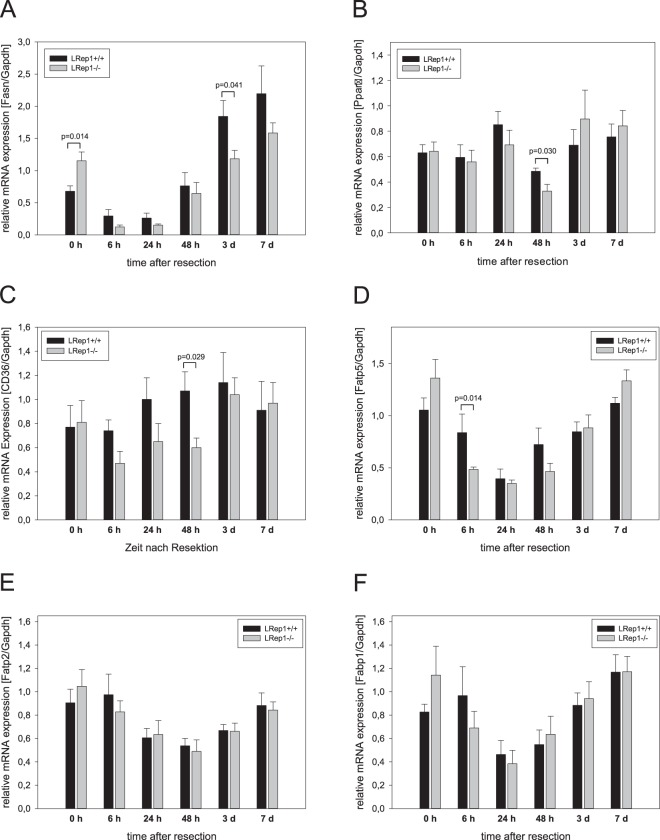
Quantitative RT-PCR analysis of (**A**) *Fasn*, (**B**) *Pparα*, (**C**) *Cd36*, (**D**) *Fatp5*, (**E**) *Fatp2* and (**F**) *Fabp1* mRNA expression in liver tissue of LRep1+/+ and LRep1−/− mice at different time points after PH (n = 6–8 per genotype and time point). Values are given as means ± SEM. Significance of differences between the groups of an individual time point was tested by Mann-Whitney Rank Sum test followed by Bonferroni correction.

In order to evaluate the impact of hepatic *de novo* lipogenesis in the process of regeneration, especially upon Repin1 deficiency, we analyzed mRNA expression of the hepatic *fatty acid synthase* (*Fasn*) as a regulatory gene essential for efficient lipogenesis (Fig. 5A). In wildtype mice *Fasn* mRNA expression transiently decreased after PH and thereafter constantly increased with values at day 3 and 7 being much higher than pre-PH (Fig. 5A). A similar kinetic could be observed in LRep1−/− livers. Because of higher basal expression, Repin1 deficiency resulted in a steeper decline of *Fasn* mRNA immediately after resection compared to wildtype mice. The extent of increase in *Fasn* expression at the later observation time points was less prominent in LRep1−/− than in wildtype mice (Fig. 5A). Additionally, we observed almost no resection-induced alterations in regulation of hepatic β-oxidation in both genotypes, as shown by almost constant *Pparα* mRNA expression, except for the time point 48 h (Fig. 5B). At that time point, *Pparα* mRNA expression was reduced in LRep1−/− compared to wildtype mice.

### Hepatic FA transport

Next, we examined the expression of different hepatic lipid transporters. Here, *Cd36* mRNA expression tended to be lower in LRep1−/− early after PH, particularly at 48 h (Fig. 5C). Also mRNA expression of the liver-specific membrane associated FA transport protein 5 (*Fatp5*) was found to be markedly diminished in LRep1−/− at 6 h (Fig. 5D), though the difference did not reach statistical significance. However, mRNA expression of other membrane associated transport proteins like *Fatp2* (Fig. 5E), intracellular FA binding proteins like *Fabp1* (Fig. 5F) or the lipid droplet fusion protein *Snap23* (data not shown) were not significantly altered between both experimental groups.

### *In vitro* analyses

**Figure 6:**
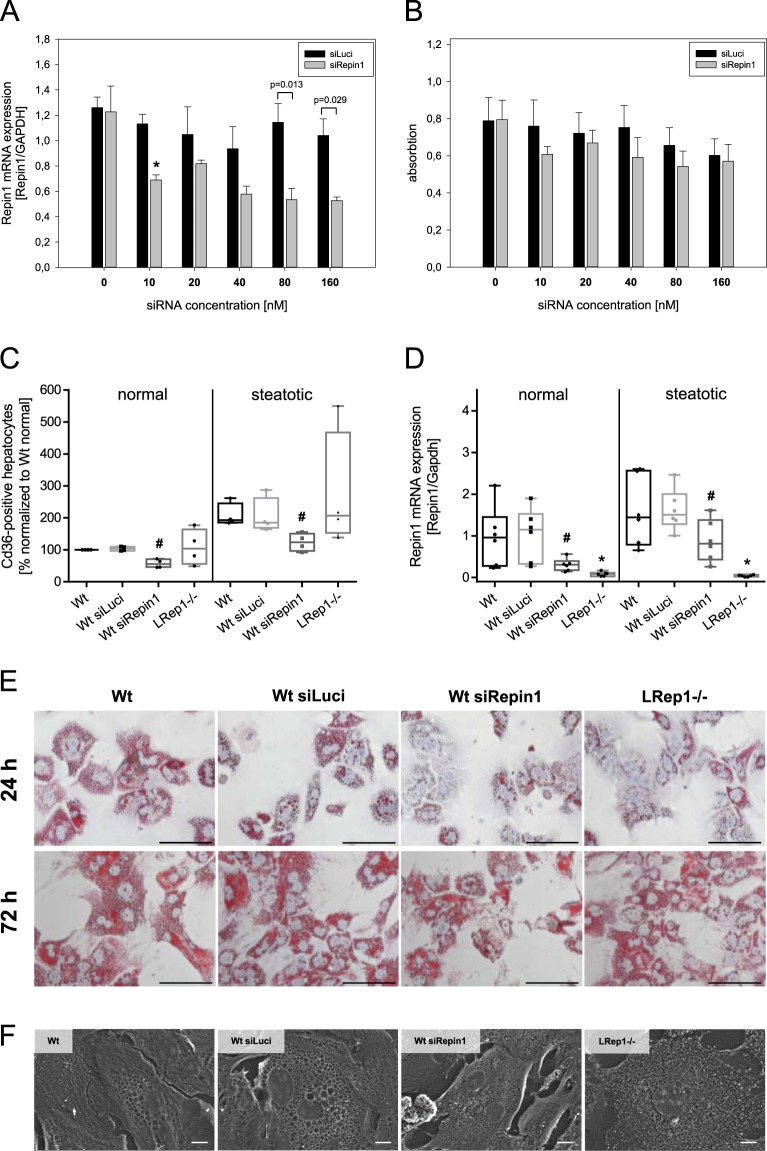
(**A**) *Repin 1* mRNA expression of HepG2 cells 72 h after treatment with 0, 10, 20, 40, 80 and 160 nM siRNA specific for Luciferase (siLuci) and Repin1 (siRepin1) (n = 3–4 per group and concentration) and (**B**) proliferation of these cells using a BrdU proliferation assay (n = 9 per group and concentration). Values are given as means ± SEM (n = 4 per genotype and time point; *p < 0.0083 vs. LRep1+/+ of the individual time point, Mann-Whitney Rank Sum test followed by Bonferroni correction. (**C**) FACS analysis of Cd36 of primary isolated wildtype hepatocytes (Wt), which additionally were transfected with siLuci or siRepin1, and of hepatocytes of LRep1−/− mice under normal (left) and steatotic (right) culture conditions for a period of 72 h. Data are presented as box plots indicating the median, the interquartile range in form of a box and the minimum and maximum as whiskers. ^#^p < 0.05 vs. Wt siLuci of the individual culture condition, Mann-Whitney Rank Sum test. (**D**) Repin1 mRNA expression of primary hepatocytes of the different genotypes and treatment groups as described above. *p < 0.01 vs. Wt; ^#^p < 0.05 vs. Wt siLuci of the individual culture condition, Mann-Whitney Rank Sum test. (**E**) Oil Red O staining of primary isolated wildtype hepatocytes (Wt), which additionally were transfected with siLuci or siRepin1, and of hepatocytes of LRep1−/− mice under steatotic culture conditions for a period of 24 and 72 h. Scale bars represent 50 µm. (**F**) Scanning and electron microscopic images of primary isolated Wt hepatocytes (Wt), which additionally were transfected with a siRNA for Luciferase (siLuci) and Repin1 (siRepin1) as well as of LRep1−/− hepatocytes after 72 h of FA exposure. Compared to controls lipid droplets (white asterisks) appear smaller in Repin1-deficient hepatocytes. Scale bars represent 10 µm.

To analyze whether Repin1 deficiency directly influences hepatocellular proliferation, we performed a BrdU proliferation assay with HepG2 cells using different concentrations of siRNA for Repin1 (siRepin1). In contrast to the nonsense siRNA for the Luciferase gene (siLuci), higher Repin1 siRNA concentrations resulted in Repin1 deficiency (Fig. 6A). However, this had no inhibiting effect on the proliferative capacity of HepG2 cells (Fig. 6B).

As CD36 is a membrane protein, we additionally performed FACS analysis of CD36 on the cell surface of primary isolated hepatocytes of LRep1−/− and wildtype mice (LRep1+/+) under normal (conventional) and, to simulate transient steatosis as in the early phase of liver regeneration, under steatotic culture conditions (Fig. 6C). Wildtype hepatocytes were also treated with 20 nM siRepin1/siLuci to evaluate the impact of a spontaneously induced Repin1 deficiency on CD36 cell surface expression. Compared to the corresponding wildtype control (Wt or Wt siLuci) *Repin1* mRNA expression was significantly reduced in primary hepatocytes of LRep1−/− mice as well as in siRepin1 treated primary wildtype cells (Fig. 6D). Generally, *in vitro* steatosis was accompanied by a slightly increased *Repin1* expression in wildtype hepatocytes (Fig. 6D). Although transfection efficiency was lower in steatotic cells compared to normal cultured hepatocytes, siRNA treatment resulted in a significantly decreased *Repin1* expression (Fig. 6D). FACS analysis of normal cultured cells revealed a significantly reduced CD36 positivity only of cells with siRNA-induced Repin1 deficiency (Fig. 6C). *In vitro* steatosis induced cell surface CD36 expression in each analyzed group of cells. A reduced CD36 expression was only observed upon spontaneously (siRNA-) induced Repin1 deficiency (Fig. 6C). Irrespective of the culture condition, CD36 expression values of LRep1−/− hepatocytes displayed a high variance. Of most interest, Oil Red O staining showed reduced lipid accumulation in both Repin1 deficient hepatocyte cultures upon FFA exposition for 24 h (Fig. 6E). However, with prolonged FFA exposure time of 72 h lipid accumulation increased with just minor differences between controls and Repin1-deficient groups (Fig. 6E).

We additionally performed SEM (Fig. 6F) and TEM (not shown) analysis of differentially cultured and treated primary hepatocytes after FA exposure for 72 h to characterize hepatocellular lipid uptake/load and intracellular lipid distribution in more detail. In general, in contrast to normal culture (not shown) *in vitro* steatosis induced formation of numerous lipid droplets of different size in wildtype cells (Fig. 6F, Wt and Wt siLuci). Noticeably, deficiency of Repin1 resulted in much smaller intracellular lipid droplets (Fig. 6F, Wt siRepin1 and LRep1−/−). TEM analysis revealed that lipid droplets are solely located intracellular (not shown).

## Discussion

Recent studies investigated distinct roles of the zinc finger protein Repin1 in adipocytes^2,4,31^. In human adipose tissue, *Repin1* mRNA expression significantly correlates with body fat content and adipocyte size^2,5^. Relationships between Repin1 expression in adipose tissue and metabolic parameters also suggest a functional role for Repin1 in the liver. As alterations in systemic and hepatic metabolism are important modulators of the physiological regenerative response to hepatic insufficiency, we therefore elucidated the potential role of Repin1 in liver regeneration after partial hepatectomy using LRep1−/− mice^6^.

The importance of Repin1 for FA uptake in adipocytes has been implied previously^2,4,6,31^ which is in line with our data, as hepatic deficiency of Repin1 causes reduced lipid content in hepatocytes during the early phase of liver regeneration. This was accompanied by a delayed cell proliferation. As fat accumulates concomitantly with cellular proliferation^32^ and Repin1 deficiency itself showed no direct effect on liver cell proliferation, it can be assumed that the reduced and shifted fat accumulation in LRep1−/− mice in turn impacts extent and course of hepatocellular proliferation, but finally without impairing liver weight recovery.

Generally, discrepant data concerning the effects of (chronic) steatosis on liver regeneration exist. However, several groups suggest that steatosis does not impair the regenerative response or even an induction of mild steatosis may be beneficial for surgical outcome of hepatectomies^30^, while others showed the opposite^24,33–35^. Much controversy continues on the predominant energy substrate (glucose or fat) for liver regeneration which complies on the availability of glucose^21^ but might primarily relate to the hepatic energy status^15,21^. Thus, with increasing resected hepatic mass and a decreasing energy charge, the liver predominantly utilizes FA as energy source^15^ and therefore transiently accumulates fat after PH^7,36^. Many studies dealing with a decreased hepatic fat accumulation imply a significant role for acute hepatic steatosis in liver regeneration^11,13,14,18,37,38^. Moreover, for several decades it has been known that infusion of lipids and carnitine following PH stimulated the initiation of liver regeneration^16,39,40^. Consistent with previous studies^19,41^ we also hypothesize that a lower temporary fat accumulation, as seen upon Repin1 deficiency, is linked to a delayed DNA synthesis in liver cells. Using different methods, we verified a significant decrease in lipids/TGs at 24 h after resection in LRep1−/− mice, while TG accumulation peaks in wildtype mice. However, LRep1−/− mice showed a compensatory shifted increase of TG at 48 h after PH. Phenotypic characterization of LRep1−/− mice^6^ suggests that reduced TG accumulation in the liver is due to decreased expression of genes involved in lipid uptake and formation. At the molecular level, we demonstrated that during liver regeneration absence of Repin1 resulted in a temporarily reduced mRNA expression of the FA transporter *Cd36* and *Fatp5*, while expression of other hepatic lipid transfer or binding genes were similar between both genotypes. FATP and CD36 have been suggested to be a functional unit^42^. Impaired and delayed FA uptake, resulting in lower hepatic TGs, was also shown in Cd36-, Fatp2- and Fatp5-deficient mice^43–46^. However, as no difference in circulating lipids (TG, FFA) was observed between both genotypes, it can also be suggested that FA transport was compensated by other mechanisms or was still sufficient to protect against dyslipidemia in LRep1−/− mice. To analyze lipid uptake and formation as well as cell surface CD36 protein expression, we additionally performed *in vitro* experiments with primary hepatocytes. In contrast to primary LRep1−/− hepatocytes, siRNA-mediated downregulation of Repin1 *in vitro* alone caused a significant reduction of cell surface CD36 expression in normal, but also steatotic hepatocytes. Interestingly, we also noticed distinct defects in efficient lipid accumulation and formation upon Repin1 deficiency by using fat staining and electron microscopy. However, differences disappeared after long-term exposure to FAs, indicating different underlying mechanisms. Hepatic FA uptake is complex and suggested to be a dual-kinetic process, consisting of a rapid, carrier-mediated, and a delayed, passive diffusional phase^37,42^. Thus, it can be supposed that Repin1 deficiency results in impairment of the facilitated, protein-mediated, phase of hepatic FA uptake (e.g. for acute conditions, like liver resection) but with preservation of the diffusional phase (e.g. upon chronic FA exposure).

Of interest, we noted that simultaneously with the onset of transient hepatic steatosis upon PH, the resection-induced liver injury is most severe. In turn, a diminished liver injury was obvious in LRep1−/−, probably due to a lower lipid load.

Due to the fact that the main source of TGs in hepatocytes during regeneration does not arise from *de novo* lipogenesis^47^, but rather from FA uptake from plasma and thus, lipolysis of systemic fat, a rise in circulating and hepatic FAs can be observed^11,38^. It is also well known, that loss of systemic adipose storages occurs in proportion to the extent of hepatic resection/insufficiency^38^. Although it is suggested that *de novo* lipogenesis provides an initial supply of FAs at the very early phase (0–6 h)^19^, expression profile of Fasn in this study indicates that *de novo* lipogenesis is not involved in the early phase of transient fat accumulation, neither in wildtype nor in LRep1−/− mice. However, Repin1 deficiency rather resulted in an impaired rise of Fasn expression in the later regenerative phase, when hepatic lipogenesis becomes more essential, suggesting a promoting role for Repin1 in lipogenesis. Interestingly, this would indicate a causal connection between the observed transient down-regulation of Repin1 and suppressed lipogenesis following PH.

By analysis of Pparα, a transcriptional regulator of FA oxidation^48^, prominent alterations in β-oxidation of FAs could not be observed during the course of liver regeneration.

Cell cycle of hepatocytes and fat accumulation are subjected to a well-defined dynamic. PH induces four waves of proliferation which are linked to three waves of fat accumulation^32^. In contrast to the DNA synthesis, which was significantly diminished in LRep1−/− hepatocytes at 48 h, mitosis index at 48 h was increased upon Repin1 deficiency. Remarkably, hepatocyte mitosis occurs consistently during a time of day determined by the circadian clock^49^. Also performing surgery between 10:00 and 12:00 am, Zou *et al*.^32^ showed that mitosis always peaks at 6:00 am, with a first mitosis peak observed at 44 h after resection. Therefore, we postulate that in this study at 48 h the mitosis peak of LRep1+/+ hepatocytes as well as a probably decreased mitosis index of LRep1−/− cells are already past and just a delayed and shifted compensatory increase in the number of mitotic LRep1−/− hepatocytes is present. In addition, the division of hepatocytes is so rapid, taking no more than 30 minutes^21^ that the mitotic index is a suboptimal indicator for the evaluation of the regenerative condition. Furthermore, as the first wave of hepatocyte DNA synthesis is present at 36–48 h after PH^10^, it also can be assumed that the differences between both genotypes are even more noticeable for earlier DNA synthesis peaks between 36 h and 44 h after PH.

Interestingly, hypoglycemia frequently occurs after partial liver resection^36^. This metabolic response is suggested to initiate signals that promote liver regeneration as dextrose supplementation suppresses PH-induced proliferation^39,50^, mainly due to inhibition of the release of free FAs from systemic adipose stores^15^. As also shown by Lai *et al*.^21^, glycogen disappeared in the early post-hepatectomy regenerative phase and reappeared constantly until day 7. Although Repin1 deficiency resulted in reduced basal glucose uptake in adipocytes due to lower Glut1 expression^2^, LRep1−/− livers restored glycogen nearly at the same degree as LRep1+/+ and showed a lower glycogen content only at day 3 after PH. To account for changes in lipid availability compensatory pathways for generation of intracellular energy sources have to be upregulated. As in LRep1−/− mice the regenerative response is just timely delayed, it can be suggested that LRep1−/− hepatocytes also use glucose as energy substrate.

Our results demonstrate that acute changes in lipid metabolism have specific effects on the course of liver regeneration with Repin1 being important for regulation of lipid load. Although hepatocytes of LRep1−/− maintain the ability to incorporate lipids after PH, however, the number of lipid droplets and finally the TG content is substantially lower than in wildtype. Consequently, insufficient amounts of energy might cause timely delayed proliferation of LRep1−/− hepatocytes. However, this had no impact on liver weight recovery which might also caused by compensatory hypertrophy^8^. As also discussed by others, a decreased expression of FA transporters seems to offer a partial explanation for a lower lipid load, but it has not clearly been determined if this effect is either due to decreased FA uptake or altered TG synthesis^51^. Besides, a more rapid turnover of TGs in the absence of Repin1 could also account for reduced TG accumulation in these mice. Vice versa, it can be assumed that reduced hepatic lipid content after resection may hamstring hepatocytes from regulating genes involved in metabolic pathways that are required for sufficient recovery. Because liver regeneration is a complex and multi-factorial process, more probably a combination of hepatic lipid load and genetic and metabolic changes determines recovery from surgery.

Due to its contribution to increased lipid accumulation and adipocyte hypertrophy, augmented Repin1 expression may play a fundamental role in adipose tissue dysfunction and its related metabolic diseases^52^. Whereas therapeutic strategies to reduce Repin1 expression are of interest in human obesity, the availability of lipids during regeneration after liver surgery is of high importance. Thus, Repin1 modulation may be a novel strategy to interfere in this process, particularly in case of limited liver regeneration of pre-existing liver injury.

## Methods

### Mice

Male mice with a hepatocyte-restricted, Cre-loxP-mediated Repin1 deletion (LRep1^AlbCre^; LRep1−/−) and wildtype littermates (LRep1+/+) (background C57BL/6 N) at an age of 10–16 weeks and with a body weight of 25–30 g were kept at a 12 h day and night cycle on water and standard laboratory chow ad libitum. Experiments were approved by the local government Landesamt für Landwirtschaft, Lebensmittelsicherheit und Fischerei Mecklenburg-Vorpommern (LALLF M-V/TSD/7221.3-1.1-099/12) and conducted in accordance with the German legislation on protection of animals and EU-directive 2010/63/EU.

### Surgical procedure and experimental groups

Mice were anesthetized by breathing isoflurane (1.5 vol%) and subjected to a 68% PH as described previously^53,54^. Sham- operated animals without hepatic resection served as group 0 h (n = 6 per genotype). The animals were allowed to recover from anesthesia and surgery under a red warming lamp and were held in single cages until the subsequent experiments followed at postoperative hours 6, 24 and 48 as well as days 3 and 7 (n = 7–8 per genotype and time point). Animals were sacrificed under ketamine/xylazine anesthesia (90/7 mg/kg bw ip) at the indicated time points (7–8 animals per time point) and blood as well as liver tissue samples were taken. The remnant livers were harvested, weighed and processed for subsequent analyses. The weight of regenerated liver was used to calculate the growth of residual liver lobes according to weight of regenerated liver/preoperative liver weight × 100 (%). As determined by sham-operated animals, preoperative liver weight was assumed as 4.7% of body weight for LRep1−/− and 5.0% of body weight for LRep1+/+. Additionally, to analyze the regenerative response, 5-bromo-2-deoxyuridine (BrdU; 50 mg/kg bw ip) was injected 1 h prior to harvest of liver tissue. BrdU incorporation into DNA was analyzed by immunohistochemistry.

### Hematological measurements and plasma enzyme levels

Blood samples were collected at final time points by retrobulbar sinus puncture. Blood cells were assessed with an automated cell counter (Sysmex KX-21, Sysmex). Activities of alanine aminotransferase (ALT), aspartate aminotransferase (AST) and glutamate dehydrogenase (GLDH) in ethylenediaminetetraacetic acid (EDTA) plasma were measured spectrophotometrically as indicators of hepatocellular disintegration and necrosis using cobas c 111 analyzer (Roche Diagnostics; Rotkreuz, Switzerland) according to the manufacturer’s instructions.

### Assays

EDTA plasma further served for the analysis of albumin as a parameter of liver function using a commercially available enzyme-linked immunosorbent assay kit in accordance with the manufacturer’s instructions (Assaypro, MO, USA). Measurements of plasma triglycerides (TG) and free fatty acids (FFA), serving as indicators of systemic dyslipidemia, were performed using the TG (Cayman Chemical Company, MI, USA; 10010303) and FFA (abcam, ab65641) assay kit methods according to the manufacturer’s instructions. Glycogen levels in liver samples were measured using a glycogen assay kit (Abnova; KA0861) according to the manufacturer’s instructions with 10 mg of homogenized frozen liver tissue. For measurement of hepatic TG, Lipids were extracted from mouse liver biopsies using the commercially available LabAssay Triglyceride (WAKO Pure Chemicals, Kyoto, Japan).

### Histopathology/Cell staining

Lipid accumulation in 8 µm frozen liver sections and cultured primary hepatocytes was visualized by Oil Red O staining. Briefly, liver sections were rinsed with 60% 2-propanol and stained for 15 min with freshly prepared and filtered Oil Red O (Sigma) solution (0,2% (w/v) in 60% 2-propanol diluted from a 0,5% (w/v) Oil Red O stock solution in 100% 2-propanol). After rinsing in 60% 2-propanol and distilled water, slides were counterstained with hematoxylin. Primary hepatocytes grown on cover slips were fixed in 4% phosphate buffered formalin (Grimm med. Logistik, Torgelow, Germany) for 5 min. After washing with PBS and water, the cells were then stained for 7 min using the same Oil Red O solution as described above. After rinsing extensively in water the cells were counterstained with hematoxylin.

Liver tissue was fixed in 4% phosphate buffered formalin for two to three days, embedded in paraffin, and cut into 5 µm thick sections. Periodic acid-Schiff (PAS) reaction was used to visualize the presence and distribution of carbohydrates, particularly glycogen, in liver cells. Therefore, slides were treated with 0.1% periodic acid solution for 5 min, washed and subsequently incubated with Schiff’s Reagent for 15 min at RT. After vigorous washing nuclei were counterstained with hematoxylin. The intensity of purple color in PAS-stained liver sections corresponds to the amount of glycogen in hepatocytes. Digital images were taken with a Color View II FW camera (Color 10 View, Munich, Germany).

### Immunohistochemistry

For analysis of DNA-incorporated BrdU in liver cells, 5 µm paraffin sections collected on poly-L-lysine-coated glass slides were incubated with monoclonal mouse anti-BrdU antibody (1:50; M0744, Dako) overnight at 4 °C followed by incubation with HRP-conjugated goat anti-mouse immunoglobin (LSAB kit plus; Dako). Sites of peroxidase-binding were detected by DAB (Dako). Sections were counterstained with hematoxylin. BrdU-positive hepatocellular and non-parenchymal nuclei (discriminated by morphology and intrahepatic location) were counted in a blinded manner within 30 consecutive high power fields (HPF) (x40 objective, numerical aperture 0.65) and are given as cells/HPF. BrdU-stained liver sections were also used to count mitotic figures (visible chromosome aggregation) in a blinded manner within 30 consecutive high power fields (HPF) (x40 objective, numerical aperture 0.65) and are given as mitotic figures/HPF.

### Conventional RT-PCR analysis

RNA isolation and conventional Real-time PCR gene expression analysis was performed as described previously^55^ using SYBR Green I (Roche, Mannheim, Germany) detection of amplified dsDNA strands applying the LightCycler System 1.5 (Roche) (Table 1). All data were calculated by using the comparative ddCt method and expression values were normalized to the expression levels of the GAPDH housekeeping gene. Target gene expression was compared to wildtype C57BL/6 N liver tissue pool.

**Table 1:** Primer sequences used for amplification by conventional quantitative real-time PCR.

Gene	Primer	Sequence (5′ to 3′)
Repin1	forward	GCCTTCTGTTGTGCCATCTGT
	reverse	TCTCAGGCATCGTGCTTCTTCC
REPIN1 (human)	forward	GAAGCAGGCGTGGTAGAGTC
	reverse	GGGAAGGAAGAGGATGGAAG
Fas	forward	TACCATGGCAACGTGACACT
	reverse	TAGCCCTCCCGTACACTCAC
Fabp1	forward	AAGTGGTCCGCAATGAGTTC
	reverse	GTAGACAATGTCGCCCAATG
Fatp2	forward	AACACATCGCGGAGTACCTG
	reverse	CTCAGTCATGGGCACAAATG
Fatp5	forward	GACTTTTGATGGGCAGAAGC
	reverse	GGGCCTTGTTGTCCAGTATG
Cd36	forward	GGTGATGTTTGTTGCTTTTATGATTTC
	reverse	TGTAGATCGGCTTTACCAAAGATG
Snap23	forward	AGAAGATCACAGAAAAGGCTGACA
	reverse	AGCAGGGCTTTAACTATCAATGAGTT
GAPDH (human)	forward	ATCACCATCTTCCAGGAGCGA
	reverse	GCCAGTGAGCTTCCCGTTCA
Gapdh	forward	GAATTTGCCGTGAGTGGAGT
	reverse	CGTCCCGTAGACAAAATGGT

### Liver lipidomics

TGs were analyzed by LC-MS on a 6550 iFunnel QTOF mass spectrometer (Agilent Technologies, Waldbronn, Germany) equipped with a Dual Agilent Jet Stream (AJS) electrospray source. Electrospray parameters were as follows: gas temperature, 200 °C; drying gas flow, 11 l/min; nebulizer pressure, 35 psig; sheath gas temperature, 350 °C; sheath gas flow, 12 l/min; capillary voltage, 4000 V; nozzle voltage, 500 V; and fragmentor voltage, 350 V. Mass spectrometric analysis was done in positive ion mode with a scan range of m/z 100–1100 and a scan rate of 4 spectra/s. Data was acquired with intensity thresholds of 10 counts/0.001% and stored in profile mode. TG standards were obtained from Larodan Fine Chemicals AB (Malmö, Sweden). Frozen liver tissue samples (about 30 mg) were homogenized in an ice cold mixture of 400 µl of methanol and 130 µl of water in a FastPrep® 24 homogenizer (MP Biomedicals, Santa Ana, USA) for 20 s at speed 6.0 using lysing matrix D. An aliquot of the homogenate was spiked with internal standard and extracted with chloroform as described previously^56^. The chloroform extract was evaporated to dryness and reconstituted in 2-propanol. Chromatographic separation of the TGs was carried out at 70 °C on a Poroshell 120 RP18 column (2.1 × 100 mm, 2.7 μm particle size, Agilent) coupled to a 1290 Infinity UHPLC System (Agilent). Gradient runs of mobile phase A (20 mM ammonium acetate in acetonitrile:water 95:5 (v/v)) and mobile phase B (20 mM ammonium acetate in 2-propanol) were programmed as follows: 0–2 min, 5–15% B; 2–4 min, 15% B; 4–8 min, 15–50% B; 8–11 min, 50–60% B; 11–12 min, 60% B, 12–13 min, 60–90% B ;13–21 min, 90% B. The column was reconditioned to initial conditions (5% B) for 5 min. TGs were analyzed as ammonium adducts [M+NH4]+ using triheptadecanoin as internal standard. Calibration samples were prepared in aqueous BSA in the concentration range from 0.0625 to 75 nmol/10 µl and were worked up as described above and analyzed together with the unknown samples. Calibration curves based on internal standard calibration were obtained by weighted (1/x) quadratic regression for the peak-area ratio of the analyte to the internal standard against the amount of the analyte. The concentration of the analytes in unknown samples was obtained from the regression curve. Assay accuracy and precision were determined by analyzing quality controls that were prepared like the calibration samples.

### Electron microscopy

For electron microscopy, hepatocytes were cultured on 13 mm circular supports cut from Melinex plastic film sheets (no. L4103, Plano, Wetzlar, Germany) with a manual punching tool. The Melinex supports were sterilized by immersion in ethanol and air-dried under a cell culture hood after washes with sterile water. For fixation, cultured hepatocytes or small blocks of liver tissue were immersed in a fixative containing 2% glutaraldehyde and 1% paraformaldehyde in 0.1 M sodium phosphate buffer pH 7.3. Prior to resin embedding for transmission electron microscopy (TEM) samples were washed three times with 0.1 M sodium phosphate buffer and were postfixed with a solution of 1% aqueous osmiumtetroxide for 1 hour. Following washes in distilled water, the cells or tissue blocks were dehydrated in a graded series of acetone completed with two pure acetone steps. Next the specimens were infiltrated with epoxy resin (Epon 812, Serva, Heidelberg, Germany) starting with 1:1 mixture of acetone and resin overnight, continued with pure resin for 4 hours and transfer to rubber molds on the following day. After curing of the resin at 60 °C for 2 days, semithin sections (0.5 µm) and thin sections (50–70 nm) were cut on a ultramicrotome (Ultracut E, Reichert&Jung, Wien, Austria) using diamond knifes (Diatome, Biel, Switzerland). Semithin sections were stained with toluidine blue to visualize the tissue structure and for further use in morphometric measurements (see below). Thin sections for ultrastructural inspection were cut from these areas, transferred to 300 mesh copper grids and were stained with lead citrate and uranyl acetate. The grids were examined in a Zeiss EM902 electron microscope (Carl Zeiss, Oberkochen, Germany) operated at 80 kV. Digital images were acquired with a side-mounted 1 × 2k FT-CCD Camera (Proscan, Scheuring, Germany) using iTEM camera control and imaging software (Olympus-SIS, Münster, Germany) with calibrated morphometric measurement tools to determine e.g. lipid droplet size and numbers.

In addition, for histomorphometric analysis of semi-thin toluidine blue stained liver sections, images of 10–15 random low power fields (10x magnification, Olympus BX 51, Hamburg, Germany) were acquired with a Color View II FW camera (Color View, Munich, Germany) and evaluated using an image analysis system (Adobe Photoshop). Fat deposition was quantified as percentage of blue stained area compared with the total section area.

For scanning electron microscopy (SEM), hepatocytes cultured on glass coverslips (no. L40971, Plano, Wetzlar, Germany) or on Melinex supports were fixed as detailed above, washed with sodium phosphate buffer and dehydrated with a graded series of ethanol or acetone. The cells were critical point dried using an Emitech K850 critical point dryer (Emitech Ltd. Ashford, UK) with CO_2_ as an intermedium. Specimens were mounted on SEM stubs with adhesive carbon tape (Plano, Wetzlar, Germany) and were coated with evaporated carbon in a Leica EM SCD 500 coater (Leica Microsystems, Vienna, Austria). The cellular structure was viewed with a field-emission SEM, Zeiss Merlin VP compact (Carl Zeiss Microscopy, Jena Germany) operated at 10 kV and digital images with a size of 1024 × 768 pixels were recorded.

### Isolation and culture of primary hepatocytes and HepG2 cell line

Hepatocytes were isolated from male LRep1−/− and LRep1+/+ mice at an age of 6 weeks as described previously^55^. Cells were plated on collagen A (200 µg/ml in PBS; Biochrom, Berlin, Germany) coated cell culture dishes with Williams E medium (PAN Biotech, Aidenbach, Germany) containing L-glutamine, 10% (v/v) FCS (for the first 4 hours, subsequently FCS free) and 100 nM dexamethasone (Sigma Aldrich) and incubated in a 5% CO_2_ humidified atmosphere at 37 °C.

The human hepatocellular carcinoma cell line HepG2 was cultured in Dulbecco’s modified Eagle’s medium (DMEM, high glucose) supplemented with 10% (v/v) FCS at 37 °C in 5% CO_2_.

### *In vitro* transfection

For siRNA transfection cells were seeded in 6-well petri dishes at a density of 1 × 10^5^ (HepG2) or 2.5 × 10^5^ (primary hepatocytes) cells per well. To transfect HepG2 cells the media was changed 24 h after seeding. Repin1 siRNAs or Luciferase siRNAs were transfected using AtuFECT01 (Silence Therapeutics, Berlin, Germany) at final siRNA concentrations of 0, 10, 20, 40, 80 and 160 nM. Primary LRep1+/+ hepatocytes were transfected simultaneously with seeding with 20 nM siRNA. After an incubation period of 4 h at 37 °C the medium was changed again and the cells were cultivated until further analyses were performed.

### BrdU-proliferation assay

HepG2 proliferation was measured by a BrdU Cell Proliferation ELISA (Roche Applied Sciences, Mannheim, Germany). For this purpose, HepG2 were trypsinized 48 h after transfection and seeded on a 96-well-plate at a density of 4 × 10^3^ cells per well. After attachment BrdU labeling solution was added and the cells were grown for additional 24 h. Fixation, staining and measurement of cell proliferation was performed according to manufacturer’s instructions.

### *In vitro* steatosis

For induction of steatosis primary LRep1−/− and LRep1+/+ hepatocytes as well as primary transfected LRep1+/+ hepatocytes were exposed to a mixture of long-chain FFA (oleic acid and sodium palmitate at a ratio of 2:1). A stock solution of 50 mM of the FFA-mixture prepared in Williams E medium containing 1% BSA was diluted in medium to obtain a final concentration of 0.5 mM. The FFA mixture was added to hepatocytes ~24 h after seeding and transfection for a period of 72 h. The medium with FFA mixture was renewed daily.

### FACS analysis

Hepatocellular cell surface CD36 expression levels were investigated by flow cytometry analyses, conducted with a FACSCalibur cytometer (BD Biosciences) running CellQuest (BD Biosciences) acquisition and analysis software. For analysis of CD36 expression primary hepatocytes were harvested after culture for 96 h, washed and incubated at 4 °C for 30 min with 20 µl anti-CD36-FITC antibody (Santa Cruz, sc-13572 FITC) or mouse IgA-FITC (sc-3900) as isotype control in FACS buffer containing PBS with 0.5% BSA. Cells were washed again and resuspended in FACS buffer for measurement. The number of CD36 positive cells (%) in each experimental group was normalized to the number of CD36 positive cells (%) of normal cultured LRep1+/+ hepatocytes.

### Statistical analysis

Results are presented as mean ± standard error of the mean (SEM) or as box plots indicating the median, the interquartile range in form of a box, and the minimum and maximum as whiskers. All statistical analyses were performed using SigmaPlot 12.0 (Systat Software Inc., Erkrath, Germany). After testing for normality and equal variance across groups differences between the two groups and time points were assessed by two-way ANOVA followed by the appropriate post hoc comparison (Holm-Sidak method) including Bonferroni probabilities to compensate for multiple comparisons, p < 0.05 was used to define statistical significance. If the data were not normally distributed, pairwise comparison was performed using Mann-Whitney Rank Sum test including Bonferroni probabilities to compensate for multiple comparisons, and thus statistical significance was set at p < 0.0083. For reasons of clarity and comprehensiveness, only statistically significant differences and p-values p < 0.05 between the groups of an individual time point are indicated in the figures.

## Data Availability

The datasets generated during and/or analyzed during the current study are available from the corresponding author on reasonable request.
